# Effects of different levels of non-pharmaceutical interventions on hand, foot and mouth disease in Guangzhou, China

**DOI:** 10.1186/s12889-022-14850-x

**Published:** 2022-12-20

**Authors:** Keyi Wu, Xiaowei Ma, Huamin Liu, Jiazhen Zheng, Rui Zhou, Zelin Yuan, Zhiwei Huang, Qi Zhong, Yining Huang, Zhoubin Zhang, Xianbo Wu

**Affiliations:** 1 Department of Epidemiology, School of Public Health, Guangdong Provincial Key Laboratory of Tropical Disease Research), Southern Medical University, Baiyun District, Nos.1023–1063, Shatai South Road, Guangzhou, 510515 China; 2 Guangzhou Center for Disease Control and Prevention, Guangzhou City, 510440 Guangdong China

**Keywords:** HFMD, COVID-19, Non-pharmaceutical interventions, Infectious disease control, Natural experiment

## Abstract

**Background:**

Non-pharmaceutical interventions (NPIs) against coronavirus disease 2019 **(**COVID-19) may have suppressed the transmission of other infectious diseases. This study aimed to evaluate the impact of different degrees of NPIs during the COVID-19 pandemic on hand, foot and mouth disease (HFMD) in Guangzhou, China.

**Methods:**

Weekly reported HFMD cases and pathogens information during 2015–2021 in Guangzhou were collected from the China National Notifiable Disease Reporting System. The observed number of HFMD cases in 2020 and 2021 was compared to the average level in the same period during 2015–2019. Then, an interrupted time-series segmented regression analysis was applied to estimate the impact of NPIs on HFMD, such as social distancing, suspension of schools, community management and mask wearing. The effects across different subgroups stratified by gender, children groups and enterovirus subtype of HFMD were also examined.

**Results:**

A total of 13,224 and 36,353 HFMD cases were reported in 2020 and 2021, which decreased by 80.80% and 15.06% respectively compared with the average number of cases in the same period during 2015–2019. A significant drop in the number of HFMD cases during time when strict NPIs were applied (relative change: 69.07% [95% confidence interval (CI): 68.84%–69.30%]). The HFMD incidence rebounded to historical levels in 2021 as the lockdown eased. The slightest reduction of HFMD cases was found among children at kindergartens or childcare centres among the three children groups (children at kindergartens or childcare centres: 55.50% [95% CI: 54.96%–56.03%]; children living at home: 72.64% [95% CI: 72.38%–72.89%]; others: 74.06% [95% CI: 73.19%–74.91%]).

**Conclusions:**

The strong NPIs during the COVID-19 epidemic may have a significant beneficial effect on mitigating HFMD. However, the incidence of HFMD rebounded as the NPIs became less stringent. Authorities should consider applying these NPIs during HFMD outbreaks and strengthening personal hygiene in routine prevention.

## Background

Hand, foot and mouth disease (HFMD) is a common acute infectious disease caused by various enteroviruses such as enterovirus 71 (EV 71) and Coxsackie virus A16 (COX A16) [1]. Children under 5 years old are the predominant population being infected with HFMD [1, 2], causing self-limiting illness, including fever, erythra and vesiculation [2]. However, some patients rapidly develop neurological and systematic complications, which are severe and fatal. HFMD can be transmitted in a variety of ways, such as direct contact with the saliva, faeces, blisters or respiratory droplets from an infected person or indirect contact with contaminated items [3]. In 2008, a large HFMD outbreak occurred in Fuyang, Anhui Province, causing 6,049 cases and 22 deaths. It raised concerns about HFMD in China. According to the outbreak, prevalence and degree of harm of infectious diseases, notifiable infectious diseases in China were classified into classes A, B and C, with class A is the worst. Medical personnel are required to report class A infectious diseases within 2 h after detection, while class B and class C infectious diseases are required to report within 24 h after diagnosis. For class A infectious disease, patients and pathogen carriers must be promptly isolated and treated, and the length of the isolation must be based on the findings of medical exams. And for class B and class C infectious diseases, appropriate steps should to be taken to treat the patient and control the disease's spread. Since 2 May 2008, HFMD was listed as a class C notifiable communicable disease, and newly HFMD cases must be reported to the National Notifiable Disease Reporting System [4].

Guangzhou is the capital of Guangdong Province, which is located in the northern edge of the Pearl River Delta. As the central city of the Guangdong-Hong Kong-Macao Greater Bay Area and the Pan-Pearl River Delta Economic Zone, Guangzhou is characterised by highly dense population [5], construction and growing economy of commerce and trading. Moreover, Guangzhou has many places for large-scale activities and people gathering, which are prone to the spread of HFMD. Epidemiological studies over the years have shown that the morbidity of HFMD in Guangzhou is higher than the national average, needing considerable attention [6]. The incidence of HFMD in Guangzhou has been increasing every year since HFMD was included as a Class C notifiable infectious diseases in 2008 [7] and a total of 30 outbreaks of HFMD are reported during 2015–2019, causing 1,957 cases with the median age of 3 years old [8]. The main site of these outbreaks is the kindergarten, which is a gathering place for susceptible people. Every year, two seasonal HFMD epidemics are reported in Guangzhou, first in late spring/summer and second in late autumn/early winter [4].

In December 2019, the coronavirus disease 2019 (COVID-19) caused by severe acute respiratory syndrome coronavirus 2 (SARS-CoV-2) emerged in Wuhan. It then quickly swept across the country and even the world, causing a worldwide epidemic. As it lacks the specific treatment against COVID-19 in the early stage of COVID-19 pandemic, countries around the world have adopted non-pharmaceutical interventions (NPIs) to prevent the spread of the epidemic [9]. China launched a first-level emergency response to public health at the beginning of the epidemic to suppress the transmission of COVID-19. The measures include social distancing, suspension of schools, encouraging employees to work at home, closed management in rural areas and communities, travel restrictions and wearing masks [10, 11]. NPIs during the COVID-19 outbreak were applied more extensively than ever before. These measures have not only decreased the spread of COVID-19, but also reduced the incidence of other infectious diseases. For instance, the reported cases of influenza [12–16], varicella, pneumonia and other respiratory infectious diseases have decreased dramatically [9, 17–20]. The transmission of dengue has also been suppressed because of social distancing during the COVID-19 outbreak [21–23]. In some regions, the cases of notifiable infectious diseases of different transmission routes decreased significantly [24–27]. For instance, respiratory diseases (e.g. influenza), intestinal infectious (e.g. infectious diarrhoea), sexually transmitted and blood-borne diseases (e.g. hepatitis B, Syphilis, AIDS) and natural focal diseases and insect-borne infectious diseases (e.g. human brucellosis) declined obviously in Guangdong Province, China. A study indicated that airborne/droplet, fecal–oral, vector-borne, and direct-contact transmitted notifiable infectious diseases reduced during COVID-19 pandemic in Taiwan.

The Guangdong government implemented different levels of emergency response to supress the spread of COVID-19 in 2020. With the control of COVID-19 epidemic, the strict lockdown was lifted, but mask wearing was still required and keeping personal hygiene (such as hand washing) was suggested. Although previous studies have demonstrated the effects of NPIs on HFMD [28, 29], they did not estimate the level and trend change of HFMD cases in different control period, and the impacts on different serotypes of HFMD were not considered. We would like to observe the effect of different degrees of NPIs on HFMD, by dividing the whole NPIs period into two segments: strict lockdown period and routine control period. Therefore, we performed an interrupted time-series (ITS) segmented regression analysis and further built our analysis by gender, children groups and different enterovirus subtype of HFMD.

## Materials and methods

### Data source

Weekly HFMD surveillance data from 1 January 2015 to 31 July 2021 in Guangzhou were obtained from the China National Notifiable Disease Reporting System [30]. A majority of HFMD cases were clinically diagnosed (the HFMD cases defined as those who had a direct or indirect contact history with infected people before illness onset, and clinical symptoms such as fever and eruption on the hands, feet, mouth, and buttocks [31]), after being diagnosed, each case should be reported to the National Notifiable Disease Reporting System within 24 h. Information for each case, including age, gender, occupation, date of illness onset and address, was obtained from the system. Each county (district) needs to perform further analysis (reverse transcription-polymerase chain reaction) on the first 5 cases of mild HFMD and the first 5 cases of severe HFMD on a monthly basis to identify specific pathogens (COX A16, EV 71 and other enteroviruses). Healthcare providers may test more patients if laboratory capacity allows. We aggregated each pathogen data into weekly counts. The data in this study is anonymous and does not involve personal privacy, thus ethics certification is not required.

Meteorological data and population data were collected from the China Meteorological Data Sharing Service system of the China Meteorological Administration (http://data.cma.cn) and the Guangzhou Statistics Yearbook (http://tjj.gz.gov.cn/), respectively.

### Lockdown during COVID-19 pandemic

**Fig. 1:**
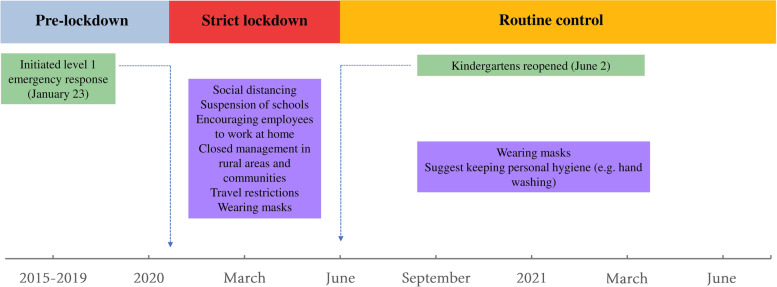
The timeline of non-pharmaceutical interventions during study period in Guangzhou, China

The emergency response in China was categorised into four levels (level 1 to level 4) based on the emergency plan for public emergencies of China, with level 1 as the highest level of response. The level 1 emergency response will initiate the most stringent public health intervention measures, such as regional lockdowns, travel restrictions, crowd bans and mandatory sanitary quarantine. The Guangdong provincial government initiated the level 1 emergency response from 23 January 2020 to 23 February 2020 to contain the COVID-19 epidemic in 2020. In addition, the level 2 response was implemented from 24 February 2020 to 8 May 2020. Since 9 May 2020, the level 3 response was implemented in Guangdong. Information about the lockdown strategy was collected from official reports of Guangzhou Municipal Health Commission [32]. Schools and kindergartens were all closed during lockdown, and the start date of the spring semester of middle school, primary school and kindergarten in 2020 was suspended to 27 April, 11 May and 2 June, respectively. The whole study was divided into three periods: pre-intervention period, strict lockdown period and routine control period. Kindergartens are known areas of high HFMD transmission, they are also common places of HFMD outbreaks [33]. Therefore, we used the end date of kindergarten suspension as the end timepoint of strict lockdown period. The pre-intervention period was from 1 January 2015 to 22 January 2020, the strict lockdown period was from 23 January 2020 to 1 June 2020, and the routine control period was from 2 June 2020 to 31 July 2021 (Fig. 1).

### Data analysis

We first performed descriptive analysis that compared the annual HFMD cases during 2015–2021. We also divided the cases into different groups by gender, age (< 1, 1–2, 3–5, ≥ 5 years), children groups (children were divided into three groups based on whether they attended school or not: living at home, at kindergartens or childcare centres, others) and enterovirus subtype of HFMD (COX A16, EV 71, other enteroviruses).

We conducted an ITS segmented regression analysis [34] to estimate the level (the y-intercept at the beginning of each segment) and trend (the slope during a segment) change of HFMD after implements of strict lockdown and routine control, and stratified the analysis by gender, children groups and enterovirus subtype of HFMD [35–37]. ITS is a strong quasi-experimental design to assess the longitudinal effects of time-limited interventions. The effects of NPIs on HFMD were compared with the counterfactual scenario that the interventions had not taken place. Allowing for over-dispersion, we applied a quasi-Poisson regression model based on the ITS design [38, 39]. The main model was presented as follows:

$$Yt\:\sim\:quasiPoisson(\mu t)$$

$$Log\lbrack E(Yt)\rbrack\;=\alpha\;+\;offset\;(\log\;\lbrack Popt\rbrack)\;+\;\beta1\;(Timet)\;+\;\beta2\;(Strict\;lockdownt)\;+\;\beta3\;(Time\;after\;Strict\;lockdownt)\;+\;\beta4\;(Routine\;controlt)\;+\;\beta5\;(Time\;after\;Rountine\;controlt)\;+\;\beta6\;(Holidayt)\;+\;\beta7\;(seasonality)\;+\;\beta8\;(weather)\;+\;\varepsilon t$$

where *t* is the sequential time in weeks from 1 January 2015 to 31 July 2021; *α* is the intercept of the model; *Y*_*t*_ and *Pop*_*t*_ are the weekly number of HFMD cases and population at time *t*, respectively. The indicator variable *Strict lockdown*_*t*_ is a binary dummy variable set to 0 during the pre-intervention period and to 1 during the stict lockdown period. The indicator variable *Routine control*_*t*_ is also a binary dummy variable, it set to 0 and 1 before and after the routine control period. In this model, *β*_*1*_ estimates the baseline trend of HFMD; *β*_*2*_ and *β*_*4*_ estimate the level change immediately after these interventions; *β*_*3*_ and *β*_*5*_ estimate the change in the long-term trend after interventions. By setting the *Strict lockdown*_*t*_* and Routine control*_*t*_ to 0 during the intervention period [40], an estimation of counterfactual scenario can be obtained. *Holiday*_*t*_ is a covariable for adjusting the potential effect of public holidays, including winter/summer school holidays. We fitted a Fourier term in the model to control the seasonality and long-term trend. We also considered the confounding effects of weather variables (including temperature and relative humidity), by using a spline function in the model. The best model was selected based on the quasi-Akaike information criterion [41]. To investigate the possible lag effects of lockdown, we conducted the model using lagged level and trend indicators. Since the incubation period for HFMD was about 3 to 7 days [4], we set the lag time as 1 to 2 weeks.

The incidence rate ratios (IRR) and 95% confidence interval (CI) were used to performed the level and trend change of HFMD cases. We further used relative change to assess the beneficial effect of NPIs on HFMD in the overall NPIs period and in two different stages (strict lockdown and routine control). The relative change was calculated by dividing the difference between the expected and predicted cases by the expected number of cases multiplied by 100%. The expected cases were estimated by the model based on the counterfactual scenario.

Plots of residuals and partial autocorrelation function were used to perform serial correlation test on the residuals of the model. And Newey-West standard errors were used to account for autocorrelation. Furthermore, we stratified the analysis by gender, children groups and enterovirus subtype of HFMD. R software (version 4.1.1) was used for all statistical analyses. Statistical significance was met for two-sided *p*-values < 0.05.

## Results

**Fig. 2:**
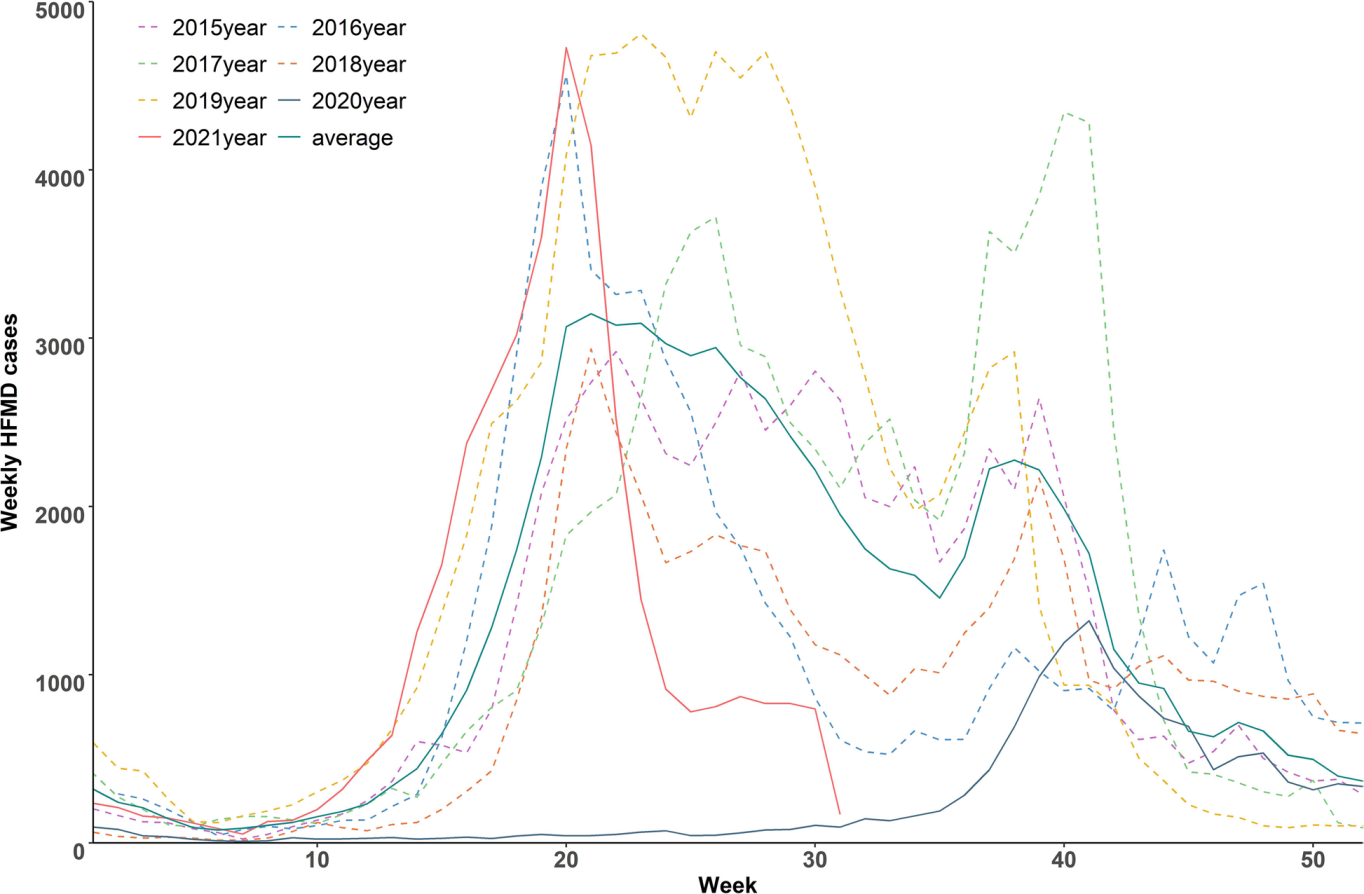
Time-series of weekly reported HFMD cases in Guangzhou, 2015–2021

As shown in Table 1, the proportion of HFMD cases were higher in male and children aged 1–2 years and children who living at home. The average number of cases in 2015–2019 was 68,872. Only 8 severe cases and 1 death case during 2015–2021 (Data not show). A total of 13,224 HFMD cases were reported in 2020, which decreased by 80.80% compared with the average annual cases in 2015–2019. When stratified by gender and children groups, the results showed that the relative reductions between gender (male: 81.93% vs. female: 79.09%) and between children groups (living at home: 81.90%; at kindergartens or childcare centres: 77.24%; others: 77.67%) were similar (Table 2). As for enterovirus subtype of HFMD, the COX A16 decreased more than the other two subtypes (COX A16: 93.99%; EV 71: 82.70%; other enteroviruses: 83.68%). The comparison between cases in 2021 and the average cases in the same period of 2015–2019 showed that, HFMD cases in 2021 decreased slightly in general (relative reduction: 15.06%). The occurrence of HFMD in Guangzhou had evident seasonality, with two epidemic peaks in May–July and September–October. In 2020, the epidemic peaks of HFMD were postponed, and the peak was lower than the average level in 2015–2019 (Fig. 2). In 2021, there was an obvious epidemic peak in the first half of year.

**Table 1:** Basic characteristics of hand, foot and mouth disease (HFMD) cases in Guangzhou from 2015 to 2021

HFMD cases	2015	2016	2017	2018	2019	2020	2021^a^
**Total cases (n)**	65,220	60,890	76,585	49,164	92,502	13,224	36,353
**Gender (n, %)**							
Male	40,003(0.613)	36,893(0.606)	46,200(0.603)	29,540(0.601)	54,476(0.589)	7485(0.566)	20,740(0.571)
Female	25,217(0.387)	23,997(0.394)	30,385(0.397)	19,624(0.399)	38,025(0.411)	5739(0.434)	15,613(0.429)
**Age (n, %)**							
< 1	9369(0.144)	5682(0.093)	11,131(0.145)	4906(0.100)	10,925(0.118)	1582(0.120)	2717(0.075)
1 ~ 2	35,776(0.549)	27,704(0.455)	38,302(0.500)	23,764(0.483)	47,063(0.509)	6384(0.483)	13,677(0.376)
3 ~ 4	17,368(0.266)	23,678(0.389)	22,714(0.297)	16,803(0.342)	27,447(0.297)	4455(0.337)	13,654(0.376)
≥ 5	2707(0.041)	3826(0.063)	4438(0.058)	3691(0.075)	7067(0.076)	803(0.060)	6305(0.173)
**Children groups (n, %)**							
Living at home	53,756(0.824)	42,831(0.703)	59,952(0.783)	35,482(0.722)	69,844(0.755)	9480(0.717)	22,677(0.624)
At kindergartens or childcare centers	9532(0.146)	15,670(0.258)	13,562(0.177)	11,229(0.228)	17,999(0.195)	3095(0.234)	11,727(0.323)
Others	1932(0.030)	2359(0.039)	3071(0.040)	2453(0.050)	4659(0.050)	649(0.049)	1949(0.054)
**Pathogens (n, %)**							
COX A16	154(0.042)	453(0.242)	167(0.080)	367(0.327)	524(0.306)	20(0.064)	223(0.311)
EV 71	520(0.141)	373(0.199)	606(0.291)	227(0.202)	299(0.174)	70(0.225)	276(0.384)
Other enteroviruses	3005(0.817)	1045(0.559)	1307(0.629)	528(0.471)	890(0.520)	221(0.711)	219(0.305)

^a^Data in 2021 was from 1 January to 31 July

**Table 2:** Comparison between the number of HFMD cases in 2020/2021 and average annual number of HFMD cases in 2015–2019 in Guangzhou

	2015–2019	2020	Relative reduction (%, 95% CI^†)^	2015-2019^b^	2021	Relative reduction (%, 95% CI^†)^
Overall	68,872	13,224	80.80 (80.50, 81.09)	42,798	36,353	15.06 (14.72, 15.60)
Male	41,422	7485	81.93 (81.56, 82.30)	25,752	20,740	19.46 (18.98, 19.95)
Female	27,450	5739	79.09 (78.60, 79.57)	17,046	15,613	8.41 (8.0, 8.84)
< 1	8403	1582	81.17 (80.32, 81.99)	4692	2717	42.09 (40.68, 43.51)
1 ~ 2	34,522	6384	85.51 (81.10, 81.92)	20,104	13,677	31.97 (31.33, 32.62)
3 ~ 4	21,602	4455	79.38 (78.84, 79.91)	13,781	13,654	0.92 (0.77, 1.09)
≥ 5	4346	803	81.52 (80.34, 82.65)	2859	6305	-120.53
Living at home	52,373	9480	81.90 (81.57, 82.23)	32,061	22,677	29.27 (28.77, 29.77)
At kindergartens or childcare centers	13,598	3095	77.24 (76.53, 77.94)	8972	11,727	-30.71 (-31.67, -29.76)
Others	2897	647	77.67 (76.12, 79.15)	1764	1949	-10.49 (-12.01, -9.14)
COX A16	333	20	93.99 (90.90, 96.08)	289	223	22.84 (18.37, 28.02)
EV 71	405	70	82.70 (78.73, 86.09)	306	276	9.80 (6.95, 13.65)
Other enteroviruses	1354	221	83.68 (81.62, 85.55)	893	219	75.48 (72.55, 78.19)

^†^*CI* confidence interval

^b^Average number of HFMD from 1 January to 31 July in 2015–2019

**Fig. 3:**
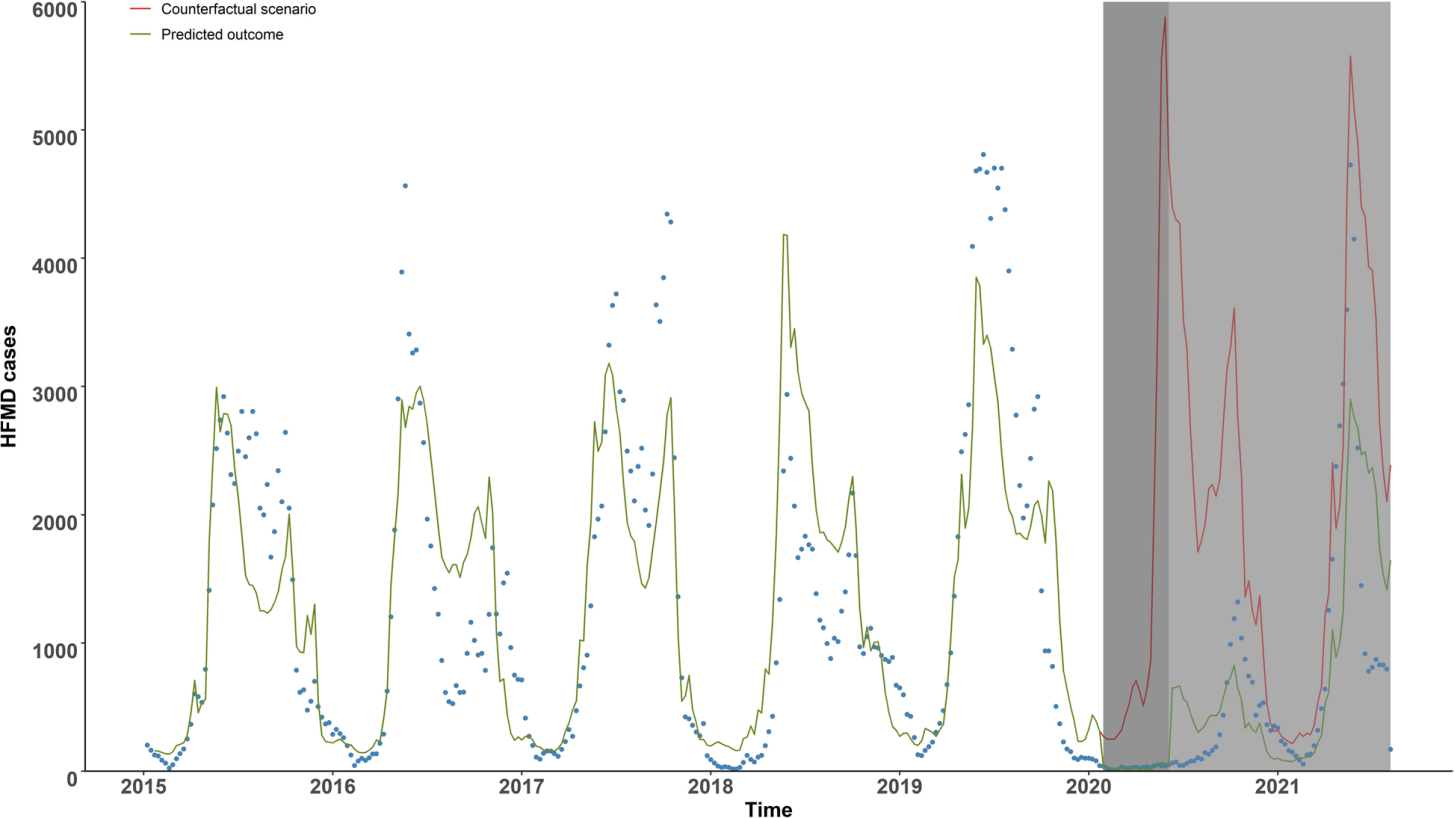
Time-series plot of observed and estimated HFMD cases from 1 January 2015 to 31 July 2021 in Guangzhou. The blue dots represent observed HFMD cases. The green line represents the predicted outcome. The red line represents the expected outcome based on the counterfactual scenario. The dark grey zone indicates the strict lockdown period and the light grey zone indicates the routine control period

In the first week of strict lockdown period, the interrupted time-series segmented regression showed that HFMD cases declined 86% compared to the no lockdown period (IRR: 0.14; 95% CI: 0.08–0.27) after controlling for long-term trend, seasonality, holiday and weather variables. After the implementation of strict lockdown, the HFMD cases decrease by a trend of 13% per week (IRR: 0.87; 95% CI: 0.83–0.90). In the second stage, when routine control measures replaced strict lockdown, the HFMD cases did not show significantly abrupt change. Whereafter, it increased by 19% per week and generated an autumn epidemic peak in 2020 (Table 3, Fig. 3). The mean temperature was positive associated with HFMD incidence (IRR: 17.94, 95% CI: 3.59–89.53), whereas relative humidity did not show significant association with HFMD incidence (data not shown). At the beginning of strict lockdown, HFMD cases in male decreased 90% while that in female decreased 80% (IRR: 0.10; 95% CI: 0.06–0.19 vs. IRR: 0.20; 95% CI: 0.11–0.38), but the trend changes during these two stages were similar between male and female (IRR: 0.88 [95% CI: 0.84–0.91] vs. IRR: 0.86 [95% CI: 0.82–0.90]). HFMD cases of children at kindergartens or childcare centres showed significant level changes in post-strict lockdown (IRR: 0.03; 95% CI: 0.01–0.10). The number of COX A16 cases dropped to zero after implementation of strict lockdown, and maintained zero COX A16 case during strict lockdown period. Cases of other enteroviruses declined by 16% per week (IRR: 0.84; 95% CI: 0.77–0.92) in strict lockdown period, whereas it raised by 19% per week (IRR: 1.19; 95% CI: 1.08–1.30) in routine lockdown period. Table 4 presents the results considering lag effects of NPIs, it is similar to the main results in general.

**Table 3:** Interrupted time-series segmented regression analysis of the impact of NPIs on HFMD at different stages

	**Level change after stage 1**	**Trend after stage 1**	**Level change after stage 2**	**Trend after stage 2**
	IRR^‡^ (95% CI)	IRR (95% CI)	IRR (95% CI)	IRR (95% CI)
Overall	0.14 (0.08, 0.27)^***^	0.87 (0.83, 0.90)^***^	2.24 (0.73, 6.90)	1.19 (1.13, 1.24)^***^
Gender				
Male	0.10 (0.06, 0.19)^***^	0.88 (0.84, 0.91)^***^	1.75 (0.55, 5.57)	1.17 (1.12, 1.23)^***^
Female	0.20 (0.11, 0.38)^**^	0.86 (0.82, 0.90)^***^	2.82 (0.92, 8.65)	1.20 (1.14, 1.26)^***^
Children groups				
Living at home	0.18 (0.09, 0.36)^***^	0.87 (0.83, 0.91)^***^	1.95 (0.60, 6.38)	1.18(1.12, 1.24)^***^
At kindergartens or childcare centers	0.03 (0.01, 0.10)^***^	0.85 (0.79, 0.92)^***^	3.40 (0.57, 20.35)	1.21 (1.12, 1.31)^***^
Others	0.25 (0.14, 0.47)^***^	0.82 (0.79, 0.87)^***^	4.40 (1.39, 13.92)^*^	1.25 (1.19, 1.32)^***^
Enterovirus subtype of HFMD				
COX A16	/ ^c^	/	/	1.28 (0.76, 2.17)
EV 71	0.30 (0.02, 5.35)	0.84 (0.63, 1.10)	8.04 (0.03, 230.27)	1.23 (0.93, 1.61)
Other enteroviruses	0.51 (0.14, 1.91)	0.84 (0.77, 0.92)^***^	11.16 (1.56,7 5.73)^*^	1.19 (1.08, 1.30)^***^

^‡^*IRR* incidence rate ratio

^*^p < 0.05

^**^p < 0.01

^***^p < 0.001

^c^HFMD cases dropped to zero and maintained at zero during the corresponding period

**Table 4:** Interrupted time-series segmented regression analysis of the impact of NPIs on HFMD with lag effects at different stages

	**Level change after stage 1**	**Trend after stage 1**	**Level change after stage 2**	**Trend after stage 2**
	IRR^‡^ (95% CI)	IRR (95% CI)	IRR (95% CI)	IRR (95% CI)
Overall (1^st^ week) Overall (2^nd^ week)	0.12 (0.06, 0.22)^***^ 0.10 (0.05, 0.19)^***^	0.87 (0.84, 0.91)^***^ 0.88 (0.84, 0.93)^***^	2.04 (0.60, 6.89) 1.94 (0.51, 7.44)	1.17 (1.12, 1.23)^***^ 1.16 (1.10, 1.22)^***^
Gender				
Male (1^st^ week) Male (2^nd^ week)	0.08 (0.04, 0.16)^***^ 0.07 (0.03, 0.13)^***^	0.88 (0.85, 0.92)^**^ 0.90 (0.85, 0.94)^***^	1.69 (0.52, 5.51) 1.39 (0.34, 5.62)	1.16 (1.11, 1.22)^***^ 1.14 (1.08, 1.20)^***^
Female (1^st^ week) Female (2^nd^ week)	0.16 (0.08, 0.31)^***^ 0.14 (0.07, 0.29)^***^	0.87 (0.83, 0.91)^***^ 0.87 (0.83, 0.91)^***^	2.44 (0.69, 8.64) 2.66 (0.39, 18.15)	1.18 (1.12, 1.24)^***^ 1.18 (1.12, 1.24)^***^
Children groups				
Living at home (1^st^ week) Living at home (2^nd^ week)	0.16 (0.08, 0.32)^***^ 0.14 (0.06, 0.28)^***^	0.88 (0.83, 0.92)^***^ 0.88 (0.83, 0.93)^***^	1.91 (0.53, 6.83) 1.93 (0.49, 7.65)	1.17 (1.11, 1.23)^***^ 1.16 (1.10, 1.22)^***^
At kindergartens or childcare centers (1^st^ week)	0.01 (0.003, 0.03)^***^	0.93 (0.86, 1.00)	0.81 (0.62, 5.69)	1.11 (1.03, 1.20)^**^
At kindergartens or childcare centers (2^nd^ week)	0.01 (0.001, 0.04)^***^	0.96 (0.85, 1.07)	0.54 (0.04, 6.81)	1.08 (0.96, 1.21)
Others (1^st^ week) Others (2^nd^ week)	0.20 (0.11, 0.36)^***^ 0.15 (0.08, 0.27)^***^	0.82 (0.78, 0.87)^***^ 0.84 (0.80, 0.88)^***^	5.20 (1.67, 16.19)^**^ 3.91 (1.07, 14.20)^*^	1.26 (1.18, 1.33)^***^ 1.23 (1.16, 1.30)^***^
Enterovirus subtype of HFMD				
COX A16 (1^st^ week) COX A16 (2^nd^ week)	/ ^c^ / ^c^	/ /	/ /	/ 1.50 (1.27, 1.95)^*^
EV 71 (1^st^ week) EV 71 (2^nd^ week)	0.08 (0.009, 6.10) / ^c^	0.90 (0.64, 1.27) /	2.07 (0.03, 157.71) /	1.13 (0.81, 1.59) 0.70 (0.45,1.09)
Other enteroviruses (1^st^ week) Other enteroviruses (2^nd^ week)	0.66 (0.19, 2.32) 0.81 (0.22, 2.96)	0.81 (0.74, 0.88)^***^ 0.78 (0.70, 0.86)^***^	25.95 (0.48, 193.60) 59.12 (0.39, 547.36)	1.23 (1.12, 1.36)^***^ 1.27 (1.14, 1.42)^***^

During the overall intervention period, 110,083 (95% CI: 107,636–112,530) cases were prevented, with a 69.07% (95% CI: 68.84%–69.30%; Table 5) reduction in number of cases. Compared with expected cases without lockdown, the reduction in strict lockdown period was greater than that in routine control period (98.01% [95% CI: 97.84%–98.17%] vs. 62.78% [95% CI: 62.52%–63.04%]). Results were similar between male and female. The least reduction of HFMD cases was found at children at kindergartens or childcare centres among the three children groups (55.50% [95% CI: 54.96%–56.03%] vs. 72.64% [95% CI: 72.38%–72.89%] vs. 74.06% [95% CI: 73.19%–74.91%]). The effect of NPIs on COX A16 cases was the greatest among Enterovirus subtype of HFMD, reaching a reduction of 73.14% (95% CI: 70.16%–75.93%). While the reductions of EV 71 and of other enteroviruses cases were slighter (EV 71: 63.09% [95% CI: 58.63%–67.34%]; other enteroviruses: 47.41% [95% CI: 44.03%–50.81%]). During the strict lockdown period, reductions between enterovirus subtype of HFMD were similar, while in routine lockdown period, COX A16, EV 71 and other enteroviruses decreased by 60.86% (95% CI: 56.97%–64.62%), 50.64% (95% CI: 45.36%–55.90%) and 35.53% (95% CI: 31.99%–39.24%), respectively.

**Table 5:** Relative change of predicted cases compared with expected cases at different stages

	**Overall**		**Strict lockdown**		**Routine control**	
	Prevented cases	Relative change (%)	Prevented cases	Relative change (%)	Prevented cases	Relative change (%)
Overall	110,083(107,636, 112,530)	69.07(68.84, 69.30)	27,907(24,169, 31,645)	98.01(97.84, 98.17)	82,176(80,257, 84,095)	62.78(62.52, 63.04)
Gender						
Male	64,455(63,022, 65,888)	69.67(69.37, 69.97)	16,252(14,091, 18,413)	98.36(98.15, 98.54)	48,203(47,063, 49,343)	63.43(63.09, 63.77)
Female	45,752(44,735, 46,769)	68.30(67.95, 68.65)	11,675(10,095, 13,255)	97.53(97.24, 97.79)	34,077(33,296, 34,858)	61.94(61.53, 62.34)
Children groups						
Living at home	84,812(83,055, 86,569)	72.64(72.38, 72.89)	18,566(16,034, 21,098)	97.35(97.11, 97.57)	66,246(64,787, 67,705)	67.82(67.53, 68.11)
At kindergartens or childcare centers	18,419(17,785, 19,053)	55.50(54.96, 56.03)	7703(6708, 8698)	99.65(99.49, 99.76)	10,716(10,288, 11,144)	42.10(41.49, 42.71)
Others	7356(7199, 7513)	74.06(73.19, 74.91)	1667(1441, 1893)	97.94(97.15, 98.51)	5689(5559, 5819)	69.12(68.11, 70.11)
Enterovirus subtype of HFMD						
COX A16	662(637, 687)	73.14(70.16, 75.93)	284(246, 322)	100.00	378(361, 395)	60.86(56.97, 64.62)
EV 71	295(283, 307)	63.09(58.63, 67.34)	122(110, 134)	96.83(92.13, 98.76)	173(167, 179)	50.64(45.36, 55.90)
Other enteroviruses	394(381, 407)	47.41(44.03, 50.81)	157(135, 179)	95.17(90.76, 97.53)	237(230, 244)	35.53(31.99, 39.24)

## Discussion

With the strict NPIs against COVID-19, HFMD in Guangzhou reached its lowest level compared with the five-year average. In this study, we assessed the effect of different degrees of NPIs on HFMD, and stratified the analysis into gender, children groups and enterovirus subtype of HFMD. The HFMD cases decreased after the implementation of strict lockdown, and increased when the intervention had converted to routine control. The overall reduction of NPIs on HFMD was 69.07% (95% CI: 68.84%–69.30%), preventing approximately 110,083 (95% CI: 107,636–112,530) cases. The impact in strict lockdown period was greater than that in routine control period. The slightest reduction of HFMD cases was showed in children at kindergartens or childcare centres among children groups.

The HFMD cases decreased dramatically during the COVID-19 outbreak, and the first epidemic peak of HFMD disappeared in the first half of 2020. The results were consistent with previous studies [28, 29]. A transmission dynamic study found that the effective reproduction number of HFMD dropped to 0 after the outbreak of COVID-19 in six cities of China [29]; and Zhao et al. indicated that NPIs against COVID-19 were associated with a substantial decrease of HFMD incidence in mainland China. A series of NPIs was released to curve the COVID-19 epidemic, including social distancing, school suspension, travel restriction, mask wearing and hand washing. These measures have not only controlled the spread of COVID-19, but also blocked the transmission of HFMD [29].

As an intestinal infectious disease, the faecal–oral route is regarded as an important route in the transmission of HFMD. In addition, hand hygiene and hygiene conditions are considered as main methods to prevent HFMD transmission [42]. Thus, strengthening hand washing may decrease the incidence of HFMD. Although school closure is a common way to contain the outbreak of infectious diseases, the lack of effective isolation may still cause the continuous prevalence of HFMD [43]. Infected children may still visit public settings such as amusement parks and shopping malls; therefore, they can transmit the virus to others [44]. In addition to school closure, the government also adopted measures such as community management, home isolation and closing of various leisure places. Consequently, the mobility of people significantly decreased after imposing the strict lockdown measure [45]. The above-mentioned measures were superimposed to prevent the transmission of HFMD through controlling the source of infection and blocking the route of transmission. Hence, the number of HFMD cases reached an unprecedented low level.

With the control of COVID-19 epidemic, the government restarted entertainment and social activities, the work resumed and schools reopened, which increased the interpersonal contacts and transmission risk of HFMD. As a result, the number of HFMD cases increased since the NPIs had switched to routine control. In the second half of 2020, an autumn epidemic peak occurred. The HFMD incidence recovered to pre-lockdown levels in the first half of 2021. This phenomenon was also found in other studies [28, 29]. In a nationwide study, Geng et al. indicated that HFMD rebounded quickly to nearly historical levels with the relaxed of NPIs, probably being the result of schools and daycares reopening [46]. In the contrast, the ongoing nationwide policy of wearing face masks in public places, may be to blame for the low incidence of respiratory diseases that persisted through the end of the year. It suggests that compared to respiratory infectious diseases, HFMD was less sensitive to some less-disruptive NPIs, such as mask-wearing. Nevertheless, a more careful personal health management including better health awareness and hygiene practices could have positive impacts on HFMD [47]. The essential elements might be to increase parental and kindergarten teachers' awareness of health and their supervisions to young children’s personal hygiene.

Stratification analysis in our study indicated that the reduction of HFMD cases in male was similar to that in female. This finding indicates that no significant difference in the beneficial effect of lockdown was observed between gender. Children living at home were the predominant group of HFMD cases in Guangzhou before lockdown [7]. Because of the lack of supervision, children living at home have poor hygiene habits and sanitary conditions [8]. Furthermore, children living at home may be infected by their siblings that at kindergartens or childcare centres. In this study, the reduction of HFMD cases decreased in routine control period relative to strict lockdown period. This decrease was more obvious among children at kindergartens or childcare centres. When kindergartens reopened, the HFMD cases among children at kindergartens or childcare centres increased greater than other two types of children in children groups. This finding indicates that the closure of schools and restriction of movements had a stronger impact on children at kindergartens or childcare centres. The decrease of HFMD cases in children living at home may be due to the strengthening of hygiene practices and sanitary conditions. Moreover, limiting outside activities may prevent exposure to pathogens and potential infected people.

The reduction of COX A16 cases was the highest both in strict lockdown period and in routine control period among all three enterovirus subtypes of HFMD. A previous study found that there were interactions between COX A16, EV 71 and other enteroviruses. The reproduction number of COX A16, EV 71 and other enteroviruses when transmit alone in the ascending period was 1.63 (95% CI: 1.47–1.79), 1.57 (95% CI: 1.42–1.71), and 1.44 (95% CI: 1.30–1.59), respectively. When EV 71 and other enteroviruses transmit together, they could decrease the reproduction number of COX A16 during the ascending period [48]. EV 71 cases and enteroviruses cases grew more than COX A16 during routine control period, probably because these serotypes of enterovirus being predominated at this period, and they transmitted together, therefore mitigated the transmission of COX A16. Moreover, maybe NPIs might have a stronger effect on COX A16 than other serotypes, but it needs more research to clarify. China has licensed EV 71 vaccine at 2016, but it has not been included in the National Immunization Program in China, which means that parents need to pay for their children's vaccinations. The basic reproduction number of EV 71 did not show significant change before and after vaccine license [33], indicating that EV 71 is still highly transmissible. Higher coverage of EV 71 vaccine needs to be promoted in addition to NPIs in the prevention of HFMD.

In summary, strict NPIs such as school closure, social distancing could effectively reduce the transmission of HFMD. During the HFMD outbreak period, closing kindergartens and improving the isolation management of infected children were important to prevent HFMD. However, closing kindergartens on such a large scale as during the COVID-19 pandemic is impractical. Strengthening personal and environmental hygiene, enhancing case identification and reporting, and increasing vaccine coverage are also critical to suppress the spread of HFMD.

This study identifies the effects of different levels of NPIs on HFMD cases whilst controlling for some time-varied biologically important covariates, such as time trend and seasonality. The study also reflected these effects in different subgroups, including gender, children groups and enterovirus subtype of HFMD. However, several limitations are shown in our study. First, the implementation of the lockdown strategy may lead to under-reporting of HFMD cases because individuals may be reluctant to leave home and seek for professional medical care. The additional burden SARS-CoV-2 places on health systems could reduce the number of non-COVID-19 patients seeking treatment, which may also result in under-reporting of HFMD cases. Second, as an ecological study, the impact of NPIs on HFMD is indirect in this study. Moreover, we could not assess individual NPIs separately and analyse the association between the COVID-19 outbreak and behavioural changes to avoid HFMD infection. Third, this model also did not consider changes in the number of people at risk due to infection immunity or any other changes, which will misestimate the effect of external interventions [35, 49]. Future studies could use alternative methods such as estimating the susceptible population using deterministic models (e.g. susceptible-infected-recovered models) and proxy indicators like vaccination rates to account for immunity. Fourth, we used the kindergarten reopening date (2 June) as the beginning of routine lockdown period, it does not exactly line up with level 1 to level 2 transition of NPIs (8 May). The effects of strict NPIs in this study may mainly due to kindergarten closure.

Our study demonstrates that the number of HFMD cases declined with implementations of rigorous NPIs during COVID-19 pandemic in Guangzhou, China. In the strict lockdown period, the HFMD cases decreased dramatically and reached an exceeding low level. After NPIs were converted to routine control, the HFMD cases increased and gradually recovered to historical levels. Hence, closing kindergartens and improving the isolation management of infected children during HFMD outbreak, and increasing parental and kindergarten teachers' supervisions to young children’s personal hygiene in routine prevention may be important to suppress HFMD.

## Data Availability

The datasets generated and/or analysed during the current study are available from the Guangzhou Center for Disease Control and Prevention. Restrictions apply to the availability of these data, which were used under license for this study. Data are available from the corresponding authors with the permission of Guangzhou Centre for Disease Control and Prevention.

## Abbreviations

HFMD: Hand, foot and mouth disease
COVID-19: The coronavirus disease 2019
SARS-CoV-2: Severe acute respiratory syndrome coronavirus 2
EV 71: Enterovirus 71
COX A16: Coxsackie virus A16
NPIs: Non-pharmaceutical interventions
ITS: Interrupted time-series
IRR: Incidence rate ratio
CI: Confidence interval
